# Intravenous mesenchymal stem cell transplantation mitigates pulmonary vascular remodeling but poses dose related risks in a pulmonary veno-occlusive disease model

**DOI:** 10.1186/s13287-025-04400-8

**Published:** 2025-05-28

**Authors:** Junko Katagiri, Jun Homma, Ryo Takagi, Hidekazu Sekine, Takeshi Shinkawa, Hiroshi Niinami, Tatsuya Shimizu

**Affiliations:** 1 Institute of Advanced Biomedical Engineering and Science, Tokyo Women’s Medical University, TWIns, 8-1 Kawada-cho, Shinjuku-ku, Tokyo, 162-8666 Japan; 2 Department of Cardiovascular Surgery, School of Medicine, Tokyo Women’s Medical University, Tokyo, Japan

**Keywords:** Pulmonary veno-occlusive disease, Pulmonary hypertension, Mesenchymal stem cells, Cell therapy, Vascular remodeling, Adverse event

## Abstract

**Background:**

Pulmonary veno-occlusive disease (PVOD) is a rare subtype of disease that causes pulmonary hypertension with vascular involvement of postcapillary structures of pulmonary vasculature. The disease has a poor prognosis with no effective therapy. The study aimed to determine whether adipose-derived mesenchymal stem cells (ASCs) alleviate pulmonary hypertension and right ventricular hypertrophy in a rat model of PVOD.

**Methods:**

Allogeneic ASCs were intravenously administered to a rat model of PVOD induced by mitomycin C. Then, muscularization in pulmonary microvessels, right ventricular systolic pressure (RVSP), and right ventricular hypertrophy were assessed using immunohistochemistry, right heart catheterization, heart weight, and hematoxylin–eosin (HE) staining. Body weight over time and survival rates were assessed.

**Results:**

ASC transplantation substantially contributed to the reduction of pulmonary microvascular muscularization in the PVOD rat model but not to the decrease in RVSP. Furthermore, it led to the attenuation of right ventricular hypertrophy and a considerable decrease in wall thickness. However, repeated ASC administration increased the mortality rate in the PVOD rat models.

**Conclusions:**

To the best of our knowledge, this is the first study to analyze the effects of ASC transplantation in a rat model of PVOD. While intravenous ASC transplantation exerts beneficial effects on the lungs and right ventricle, adverse events may occur depending on the administration method. Therefore, intravenous ASC transplantation should be performed with caution.

**Supplementary Information:**

The online version contains supplementary material available at 10.1186/s13287-025-04400-8.

## Introduction

Pulmonary veno-occlusive disease (PVOD) is a pulmonary hypertension disorder that is characterized by an increase in mean pulmonary arterial pressure of 25 mmHg or greater at rest. Pulmonary hypertension has a poor prognosis owing to right ventricular failure provoked by persistent growth of pulmonary arterial pressure [1, 2]. PVOD is recognized as a rare subtype of pulmonary hypertension, characterized by pathological changes involving arteries, veins, and capillaries; furthermore, its pathophysiology of this condition is poorly understood [3, 4]. Clinically, the use of alkylating agents, such as mitomycin C (MMC), a chemotherapeutic drug, has been associated with the development of PVOD [5]. MMC-induced PVOD is a life-threatening condition due to its severe and rapidly progressive nature, and it presents unique challenges in management, including a potential risk of pulmonary edema with conventional pulmonary hypertension therapies [5]. To date, no well-established therapy for PVOD exists, with lung transplantation being the only mode of treatment [1]. Investigation of treatment for PVOD showed that MMC promoted PVOD development in rats as animal models and that amifostine, a radioprotective agent, was effective in ameliorating right ventricular hypertrophy and improving survival in rats [6]. However, it has not been clinically used owing to its toxicity [7].

Since the late 1990s, regenerative medicine using cell transplantation has gained considerable attention, with mesenchymal stem cells (MSCs) in particular being recognized for their anti-inflammatory, enhanced antiapoptotic, and immunomodulatory roles in tissue repair under various pathological conditions [8]. Furthermore, MSCs have been considered to be a promising cell source as they can be easily isolated from multiple sources, and immune tolerance to them enables allogeneic transplantation [9]. In light of these advantages, clinical trials using MSCs for refractory diseases, such as respiratory, neurological, and musculoskeletal disorders, have been conducted [10]. In addition, emerging evidence indicates that MSC transplantation for pulmonary hypertension is promising [11]. However, to the best of our knowledge, there have been no reports documenting the administration of MSCs in the MMC-induced PVOD model. Consequently, the present study aimed to investigate the efficacy and safety of allogeneic MSC transplantation for PVOD.

The regenerative therapeutic effect of MSCs had been thought to be caused by paracrine factors rather than the differentiation of engrafted MSCs into a damaged tissue [9, 10]. The detected timing of MSC transplantation that enables harnessing of the effect of paracrine varies across studies. In the context of myocardial infarction, the cardioprotective effect of MSCs was observed as early as 72 h postinfarction [9]. Then, as regards the therapeutic effects, a previous study involving rat models of PVOD reported right ventricular hypertrophy due to pulmonary hypertension and did not demonstrate substantial differences until week 4 [12], suggesting that the effects were analyzed at later time points. Herein, we decided to administer MSCs on day 10 after the initial MMC injection to enable assessment of the therapeutic outcome weeks after the injection. In addition, we evaluated the potential impact of administering two intravenous doses of MSCs at spaced intervals on the therapeutic outcome was investigated.

The present study aimed to investigate the effects of allogeneic MSC transplantation on the lungs and its role in the alleviation of pulmonary hypertension in a rat model of PVOD. We also studied whether they contribute to the amelioration of right ventricular hypertrophy.

## Materials and methods

### Ethical statement

All animal experiments were conducted according to the protocols approved by the Ethics Committee for Animal Experiments of Tokyo Women’s Medical University (approval number: AE23-120, AE24-074) and the ARRIVE guidelines 2.0. All animals were placed in separate cages and provided with food and water *ad libitum* under a 12-h light cycle as well as constant temperature and humidity. The humane endpoint was defined as when the rats became emaciated to the point of losing more than 20% of their pre-experimental body weight, their general condition deteriorated, or they became substantially less active, in which case the experiment was immediately terminated, and the rats were euthanized by inhalation anesthesia with 5% isoflurane followed by exsanguination.

### Study design

**Fig. 1:**
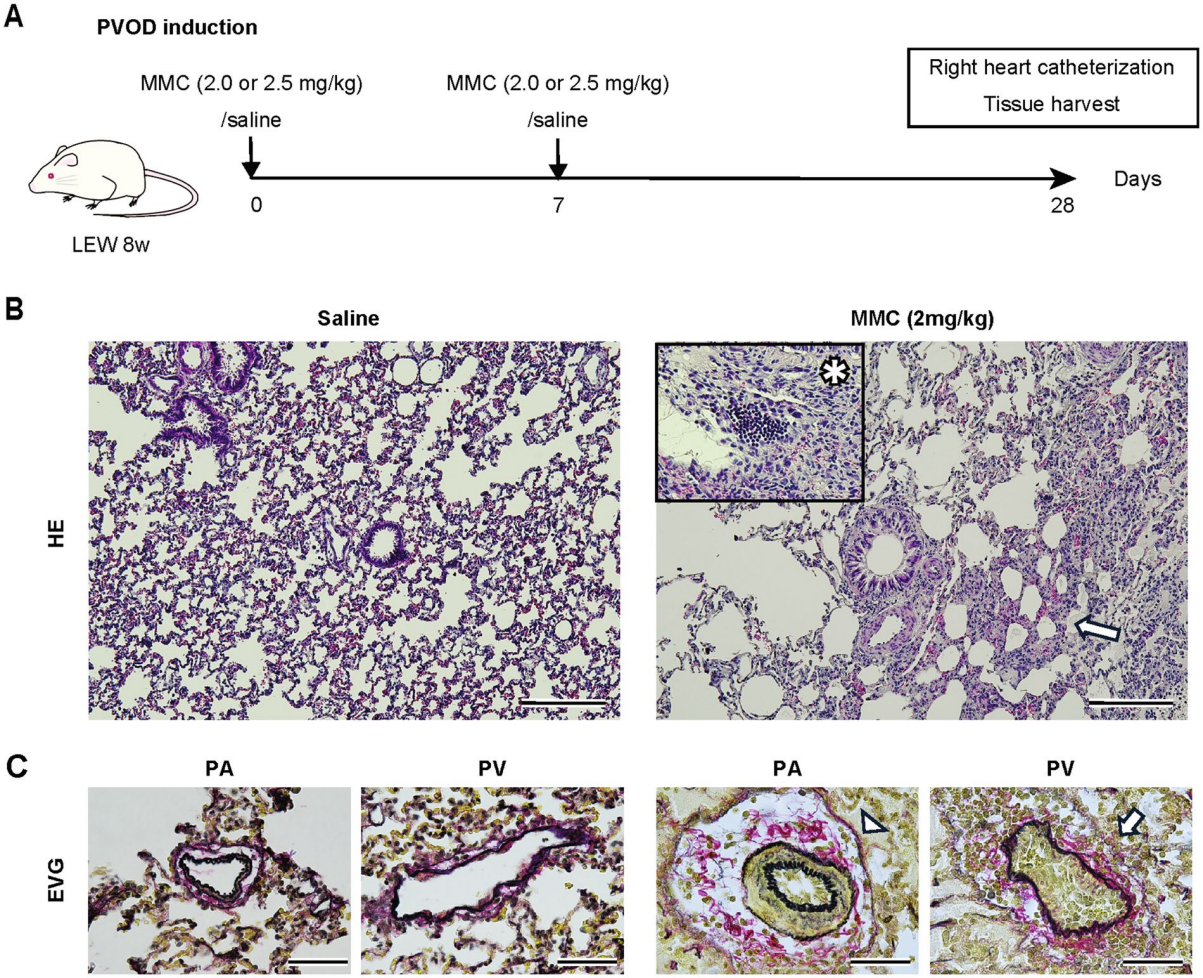
Mitomycin C (MMC)-induced pulmonary veno-occlusive disease (PVOD) in rats. **A**, Schematic of the PVOD induction experiment. Rats were intraperitoneally administered with 2 or 2.5 mg/kg of MMC on days 0 and 7 (*n* = 10 per group). The control group was injected with saline (saline group, *n* = 15). **B**, Representative images of HE-stained lung tissues from each group. The surrounding square in the right image denotes an enlarged image of MMC. MMC-exposed lungs exhibited inflammatory cell infiltrates and alveolar wall thickening (arrows and asterisk). Scale bar: 200 μm. **C**, The EVG-stained images showed luminal obstruction of the vein (arrows) and medial hypertrophy of the artery (arrowheads) in the MMC-exposed lungs. Scale bar: 50 μm. MMC, mitomycin **C**; PVOD, pulmonary veno-occlusive disease; HE, hematoxylin and eosin; EVG, Elastica van Gieson

Male 8-week-old Lewis rats (Japan SLC, Shizuoka, Japan) were used in the transplantation experiments, and male 4-week-old Lewis rats (Japan SLC) were used to harvest adipose-derived stem cells (ASCs). In the PVOD induction experiments, the 8-week-old rats were randomly selected and intraperitoneally injected with mitomycin C (MMC; Tokyo Chemical Industry, Tokyo, Japan) or saline as a control (saline group) on days 0 and 7, according to a previous study [6] (Fig. 1A). In the ASC transplantation experiments, the MMC-exposed rats were intravenously injected with ASCs (MMC + ASC group) or phosphate-buffered saline (PBS; MMC group) on day 10. The rats that had been administered with saline were also injected with the same volume of ASCs (saline + ASC group) on day 10. At week 4, the rats underwent right heart catheterization under inhalation anesthesia with isoflurane and were sacrificed for evaluation (Fig. 3A). The statistical power and sample size were calculated with reference to previous studies [6] and by utilizing G*Power 3.1.9.7 software (Heinrich-Hein-University of Düsseldorf, Düsseldorf, Germany). The total number of animals used was 106. The two rats in the MMC + ASC group were not included in the analysis because they died early due to MMC toxicity prior to ASC transplantation (prior to treatment intervention).

### Isolation and culture of ASCs

ASCs were isolated from rat inguinal adipose tissues, as previously reported [13, 14]. Five rats were used for ASC isolation. All cells were harvested using DMEM/F12 medium (Thermo Fisher Scientific, Waltham, MA) supplemented with 10% fetal bovine serum (FBS), 1% penicillin-streptomycin (FUJIFILM Wako, Tokyo, Japan), and 0.2-mM L-ascorbic acid phosphate magnesium salt n-hydrate (FUJIFILM Wako, Tokyo, Japan) at 37 °C in 5% CO_2_.

### Preparation of ASCs for transplantation

To ensure consistency in the properties of ASCs used for treatment, ASCs at passage 1 were cryopreserved at -80 °C. Before administration, they were thawed, seeded onto culture dishes, and expanded. ASCs at passage 2 or 3 were harvested using Accutase (Innovative Cell Technologies, San Diego, CA) and suspended in PBS at a concentration of 1 × 10^6^ cells/mL. The rats were anesthetized with isoflurane, incised in the inguinal region, and administered with ASCs (1 × 10^6^ cells) via the femoral vein using a 24-gauge syringe. After compression hemostasis, the wound was closed.

### Flow cytometry

The cells cultured as previously described were incubated with antibodies (Table S1) for 1 h at room temperature and then rinsed with PBS containing 2% FBS. Subsequently, the cells were subjected to a nylon mesh filtration process. All the samples were then analyzed using a Gallios flow cytometer (Beckman Coulter, Brea, CA), and data were evaluated using the Kaluza Analysis software version 2.1. (Beckman Coulter, Brea, CA).

### Tracking of intravenously administered ASCs

ASCs labeled with a CellTracker™ Green CMFDA fluorescent probe (Thermo Fisher Scientific, Waltham, MA) were intravenously injected into healthy Lewis rats according to the manufacturer’s protocols. In the sham operations, an equivalent volume of PBS was injected. Approximately 4 h after the injection, the rats were euthanized by inhalation anesthesia with 5% isoflurane, and lung, liver, and spleen were harvested. Images were obtained using the MVX10 fluorescence stereomicroscope (OLYMPUS, Tokyo, Japan).

### Right heart catheterization and sample collection

The rats were anesthetized with isoflurane, and a J-shaped polyethylene catheter (Primetech Corporation, Tokyo, Japan, BTPE-50) was inserted into the right ventricle via the right internal jugular vein. Hemodynamic data were recorded and analyzed using the PowerLab 4/30 system and LabChart software (AD Instruments, Dunedin, New Zealand). As the procedural duration for right heart catheterization was kept brief, not exceeding one hour, the procedure was carried out at room temperature with the rat’s body surface adequately covered in gauze to maintain body temperature. After pressure measurement, blood was obtained via the jugular vein, allowed to clot at room temperature for 2 h, and then centrifuged at 1,500 *g* for 15 min. Then, supernatant serum was collected. A chest cavity was accessed, the organs were perfused with saline, and heart and lungs were harvested. The Fulton index was calculated as the ratio of right ventricular weight to left ventricular and septal weights and was used as an indicator of right ventricular hypertrophy [6, 15].

### Histology

Left lung lobes and right ventricle tissues were fixed in a 4% buffered paraformaldehyde solution. The samples were embedded in paraffin, sliced into 5-µm thick sections, and subjected to staining with hematoxylin–eosin (HE), Elastica van Gieson (EVG), and picrosirius red. All images were obtained using the ECLIPSE Ci-L Plus microscope (NIKON, Tokyo, Japan). The wall thickness of the HE-stained right ventricular tissue was evaluated at 10 locations per section, and the average value was determined. Furthermore, using ImageJ version 1.54 f (National Institutes of Health, Bethesda, MD), the percentage of collagen area was evaluated from five randomly selected fields of view in each picrosirius red-stained right ventricular tissue.

### Immunohistochemistry

The paraffin-embedded samples were sectioned at 5-µm thickness, deparaffinized, and rehydrated. After heat-induced antigen retrieval and blocking, the sections were incubated with primary antibodies against CD31 (Abcam, ab281583) and α-smooth muscle actin (αSMA, Dako, M0851) at 4 °C overnight and then with secondary antibodies (Alexa Fluor™ 488 Goat Anti-Rabbit IgG, Thermo Fisher Scientific, A11008; Alexa Fluor™ 568 goat Anti-Mouse IgG, Thermo Fisher Scientific, A11019) for 45 min at room temperature in the dark. The sections were finally mounted with ProLong™ Gold Antifade Mountant with DAPI (Thermo Fisher Scientific, P36935). Images were then obtained using the BZ-X800 fluorescent microscope (Keyence, Tokyo, Japan). Six fields of view were randomly selected from each lung section, and pulmonary microvessels measuring 20–100 μm in each field were analyzed for the αSMA area in the lumen using ImageJ version 1.54 f (National Institutes of Health, Bethesda, MD).

### Enzyme-linked immunosorbent assays

The concentration of N-terminal pro-brain natriuretic peptide (NT-proBNP) in serum was determined using rat NT-proBNP ELISA kits (Biomedica, BL-1204R) according to the manufacturer’s protocols. Duplicate assays of all the samples were also performed.

### Statistical analysis

Descriptive statistics were expressed as mean ± standard error of the mean (SEM). Two-tailed Student’s *t*-tests, Pearson’s correlation, and one-way analysis of variance (ANOVA) were employed as appropriate after confirming the normal distribution of the data using the Kolmogorov–Smirnov test. Nonparametric tests were employed for non-normally distributed data. The specific test used was presented in the associated figure legend. Survival curves were obtained using the Kaplan–Meier method and comparatively analyzed using the log-rank test. Statistical analyses were conducted using GraphPad Prism 10.2.3 and R 2.7-1. *P* < 0.05 was considered to indicate statistical significance.

## Results

### Dose adjustment of mitomycin C resulted in the successful establishment of the PVOD rat model

First, as shown in Fig. 1A, the PVOD rat model was established through the administration of mitomycin C in accordance with the referenced study [6]. At the 2.5-mg/kg dose of MMC, 4 of 10 rats were sacrificed owing to the toxicity observed by the end of the course. Thus, for ethical reasons, the dose was set to 2 mg/kg. The rats administered with 2 mg/kg of MMC weighed substantially less on day 28 than those in the control group; however, their food intake slightly reduced on day 28 (Fig. S1). All rats administered with 2 mg/kg of MMC survived until evaluation. Accordingly, subsequent experiments were conducted with an evaluation at 2 mg/kg of MMC.

Histological evaluation showed that MMC-exposed lungs exhibited alveolar wall thickening and inflammatory cell infiltrates in the lung parenchyma (Fig. 1B). The characteristic findings of PVOD were medial hypertrophy of the pulmonary arteries, which were anatomically identified by being adjacent to the bronchioles, and pulmonary vein obstruction (Fig. 1C). The right ventricular systolic pressure (RVSP) substantially increased to 39.8 ± 3.2 mmHg in rats administered with MMC, whereas it increased to 28.9 ± 0.7 mmHg in the control group. An increase in the Fulton index (i.e., RV/LV + S) was also observed between the groups (38.5% ± 2.8% vs. 26.1% ± 0.9%; Fig. S2).

### Pre-labeled ASCs were found in rat lungs

**Fig. 2:**
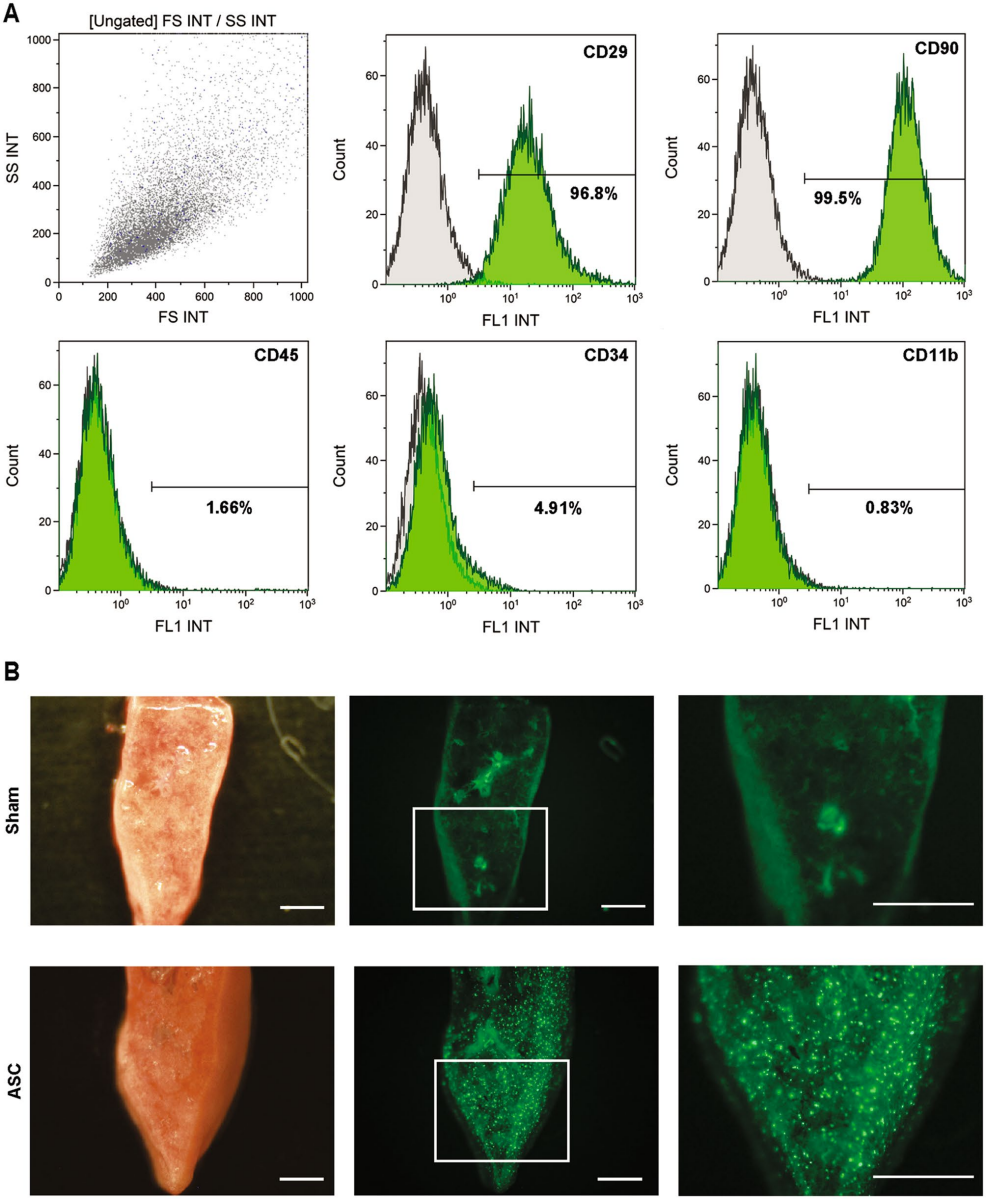
Characterization and tracking of ASCs after intravenous administration. **A**, ASCs at passage 2 were incubated with antibodies against each surface marker (green) and the corresponding isotype control (gray) and then analyzed via flow cytometry. CD29 and CD90 were positive, whereas CD45, CD34, and CD 11b were negative. **B**, Approximately 4 h after intravenous administration of green fluorescence-labeled ASCs (1 × 10^6^) to healthy rats via the femoral vein, a uniform distribution of ASCs was observed in the lungs. In sham surgery, the same volume of PBS was administered. Scale bar: 1 mm. ASCs, adipose-derived mesenchymal stem cells

Next, the cells used in the experiment were qualitatively described. The cells had a spindle-shaped morphology (Fig. S3). Flow cytometry analysis revealed that CD29 and CD90 were positive, whereas CD45, CD34, and CD11b were negative (Fig. 2A), consistent with the previously reported rat mesenchymal stem cell markers [13]. To confirm the distribution of ASCs in specific organs, ASCs pre-labeled with green fluorescence were intravenously injected to healthy rats, and then the rats were sacrificed to observe the organs. Approximately 4 h after, the diffuse spread of ASCs was detected in cross-sections of the lungs (Fig. 2B), with no obviously fluorescent cells in the liver and spleen connected to the systemic circulation (Fig. S4).

**Fig. 3:**
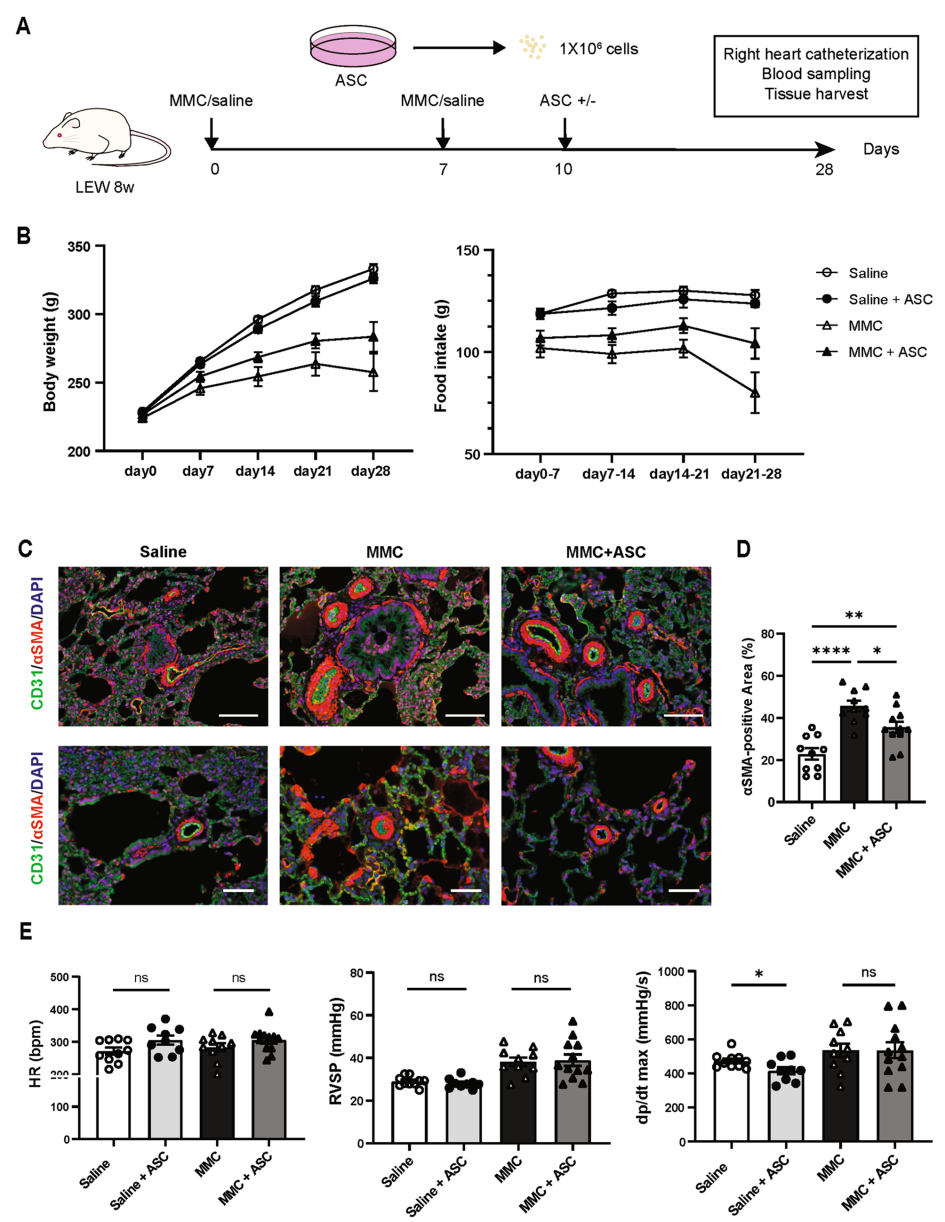
ASC transplantation inhibited the muscularization of pulmonary microvessels in the PVOD rat model. **A**, Schematic of the ASC transplantation experiment for the PVOD rat model. **B**, Body weight and food intake during the course of the study in the four groups (saline group, *n* = 15; saline + ASC group, *n* = 10; MMC group, *n* = 15; MMC + ASC group, *n* = 16). **C**, Representative images of rat lung immunostaining. Scale bar (top): 100 μm. Scale bar (bottom): 50 μm. **D**, Quantitative evaluation of the αSMA-positive area in the pulmonary microvessels (saline group, *n* = 10; MMC group, *n* = 10, MMC + ASC group, *n* = 11). One-way ANOVA followed by Tukey’s multiple comparison test was employed. **E**, Summary of right heart catheterization outcomes at week 4 (saline group, *n* = 10; saline + ASC group, *n* = 9; MMC group, *n* = 10; MMC + ASC group, *n* = 12). Comparisons between the saline vs. saline + ASC and MMC vs. MMC + ASC groups were performed using unpaired *t-*test. All the data were expressed as mean ± SEM. *P < 0.05. αSMA, α-smooth muscle actin; DAPI, 4’,6-diamidino-2-phenylindole; HR, heart rate; RVSP, right ventricular systolic pressure

### ASC transplantation reduced muscularization in pulmonary microvessels

To examine the therapeutic effect of ASCs, PVOD-induced rats were transplanted with ASCs on day 10 (Fig. 3A). The body weight and food intake of the MMC + ASC group increased on day 28 compared with the MMC group (body weight, 284 ± 11 vs. 258 ± 14 g, *P* = 0.15; food intake, 104 ± 8 vs. 80 ± 19 g, *P* = 0.08; Fig. 3B). The body weight or food intake of the saline + ASC group was not altered compared with that of the saline group (Fig. 3B). The immunohistochemical evaluation of lungs revealed that the pulmonary microvessels of the MMC group showed muscularization by αSMA-stained cells (Fig. 3C). The positive area of αSMA in the pulmonary microvessels considerably increased in the MMC group than in the saline group. However, the positive area of αSMA in the pulmonary microvessels in the MMC + ASC group was considerably lower than that in the MMC group (Fig. 3D). The catheterization results indicated that the RVSP of the MMC + ASC group did not significantly change compared with that of the MMC group. Meanwhile, the RVSP of the saline + ASC group was comparable to that of the saline group. The dp/dt max of the saline + ASC group was substantially lower than that in the saline group (Fig. 3E).

### ASC transplantation ameliorated right ventricular hypertrophy

**Fig. 4:**
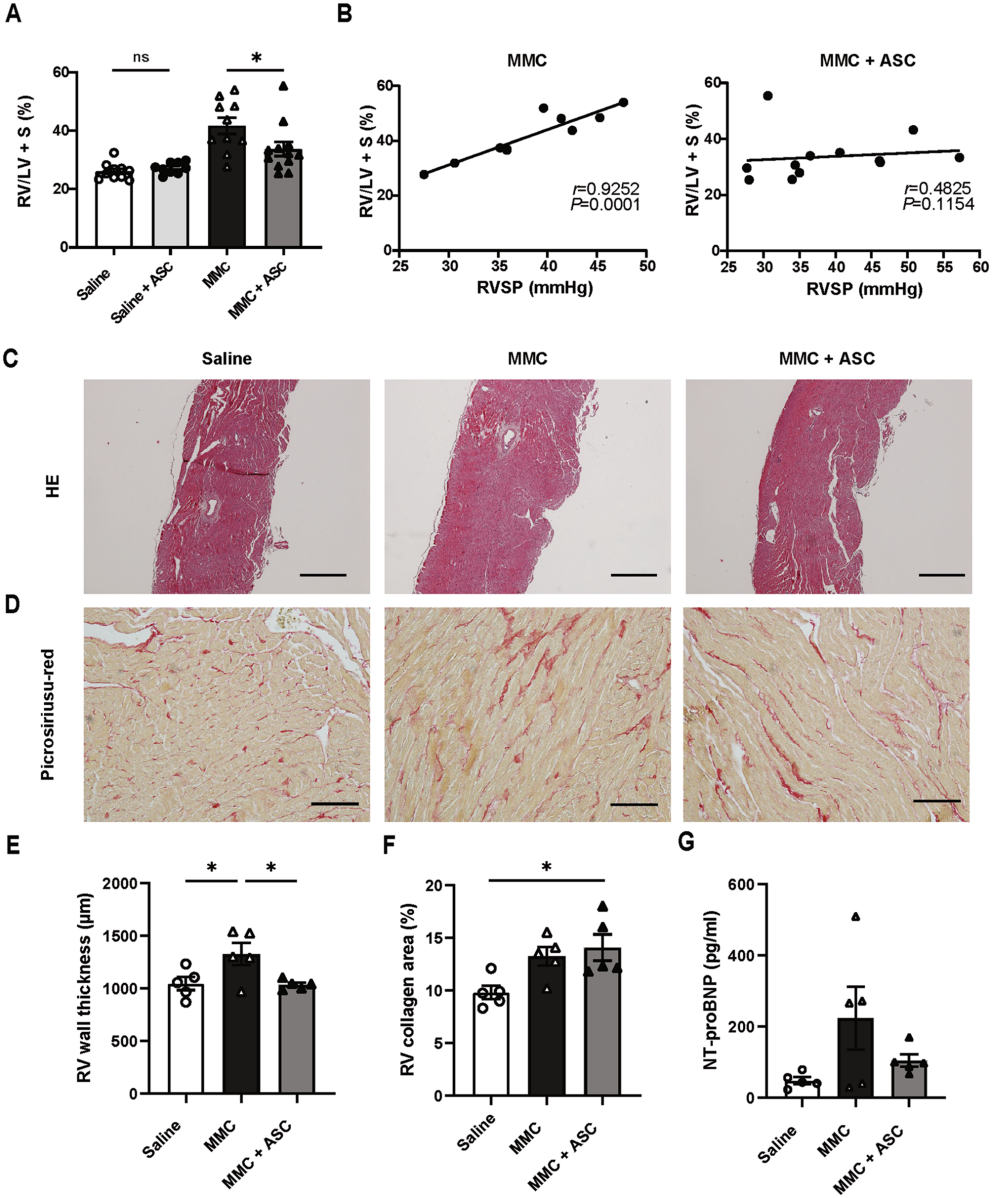
ASC transplantation mitigated right ventricular hypertrophy in the PVOD rat model. **A**, Quantification of the Fulton index (i.e., RV/LV + S) (saline group, *n* = 10; saline + ASC group, *n* = 9; MMC group, *n* = 10; MMC + ASC group, *n* = 12). Unpaired *t-*test was employed for the comparative analysis between the saline and saline + ASC groups and Mann–Whitney *U*-test for the analysis between the MMC and MMC + ASC groups. **B**, Correlation analysis between the RVSP and Fulton index. Pearson’s correlation test was employed for the MMC group (*n* = 10) and Spearman’s rank correlation test for the MMC + ASC group (*n* = 12). **C**, Representative images of HE-stained right ventricular tissue. Scale bar: 500 μm. **D**, Representative images of picrosirius red-stained right ventricular tissue. Scale bar: 100 μm. E, Quantification of right ventricular wall thickness (saline group, *n* = 5; MMC group, *n* = 5; MMC + ASC group, *n* = 5). One-way ANOVA followed by Tukey’s multiple comparison test was conducted. **F**, Quantification of the collagen area in the right ventricular tissue (saline group, *n* = 5; MMC group, *n* = 5; MMC + ASC group, *n* = 5). One-way ANOVA followed by Tukey’s multiple comparison test was employed. **G**, Quantification of serum NT-proBNP at week 4 (saline group, *n* = 5; MMC group, *n* = 5, MMC + ASC group, *n* = 5). All descriptive statistics are expressed as mean ± SEM. *P < 0.05. RV/LV + S, ratio of right ventricle weight to left ventricle plus septum weight; NT-proBNP, N-terminal pro-brain natriuretic peptide

To evaluate right ventricular hypertrophy, the ratio of right ventricular weight to left ventricular and septal weights was established as the Fulton index. The Fulton index value of the MMC + ASC group was considerably lower than that of the MMC group (Fig. 4A). Pearson’s correlation revealed a positive correlation between the RVSP and Fulton index in the MMC group but not in the MMC + ASC group (Fig. 4B). As expected from the Fulton index evaluation, quantitative analysis of the right ventricular wall thickness revealed a substantial increase in values in the MMC group compared with the saline group; however, this effect was attenuated by ASC transplantation (Fig. 4C and E). To determine whether a higher right ventricular weight ratio was caused by right ventricular fibrosis, the collagen area of a picrosirius red-stained right ventricular tissue was evaluated. This value was substantially higher in the MMC group than in the saline group but comparable to that in the MMC + ASC group (Fig. 4D and F). NT-proBNP, a biomarker of right ventricular dysfunction [16], was measured in serum at week 4 and was not found to be higher in the MMC + ASC group than in the MMC group (105 ± 14.2 vs. 223.9 ± 72.4, *P* = 0.28; Fig. 4G).

### Two injections of ASCs decreased the survival rate of the PVOD rat model

**Fig. 5:**
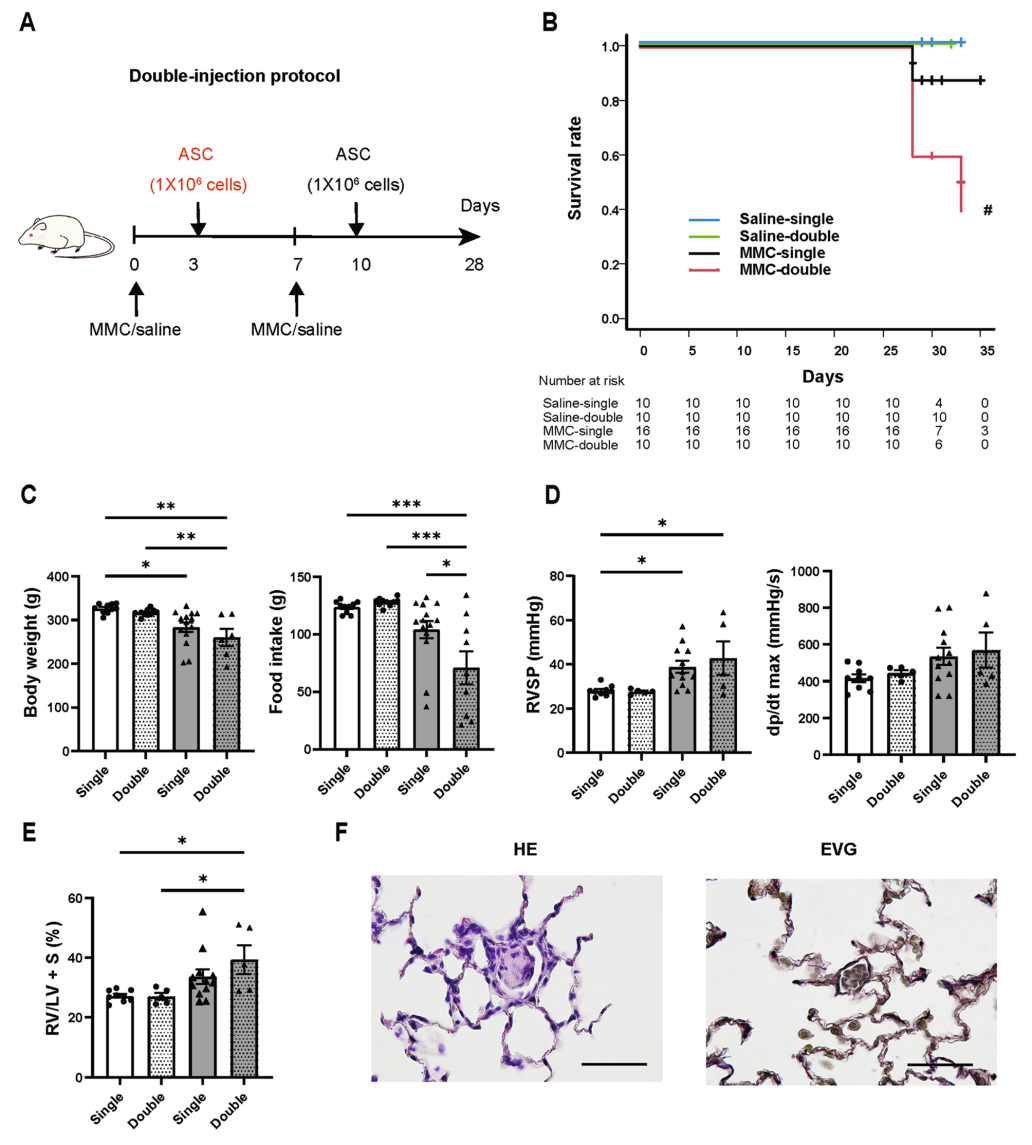
The double-injection treatment for the PVOD rat model decreased the survival rate of rats compared with the single-injection treatment. **A**, Schematic of the double-injection protocol. ASCs were transplanted in rats on days 3 and 10. **B**, Kaplan–Meier survival analysis results for single or double injections administered to saline rats or MMC rats. The global log-rank test showed a significant difference; therefore, pairwise comparisons were conducted without applying multiplicity correction (saline-single group, *n* = 10; saline-double group, *n* = 10; MMC-single group, *n* = 16; MMC-double group, *n* = 10). **C**, Comparison of body weight and food intake on day 28 among four groups: saline rats and surviving MMC rats subjected to single or double injections. **D**, Catheterization results at week 4 among the four groups. **E**, The Fulton index results across the four groups. **F**, The lung tissue images from the MMC rats, taken the day after the double-injection protocol, revealed emboli composed of cell aggregates in the pulmonary microvasculature (*n* = 3). Scale bars: 50 μm. **C** to **E**, One-way ANOVA followed by Tukey’s multiple comparison test was performed. *P < 0.05, **P < 0.01, ***P < 0.001. ^#^*P* < 0.05 vs. saline-double group. Note: Here, the saline-single group and MMC-single group correspond to the saline + ASC group and MMC + ASC group, respectively

Finally, to investigate whether administering a greater number of cells could enhance the therapeutic effects on the lungs and heart, ASC administration was performed twice in both saline rats and MMC rats. On day 3, 1 × 10^6^ ASCs were injected, and the same dosage was repeated on day 10 (Fig. 5A). Consequently, in the MMC rats receiving two ASC injections (MMC-double group), 5 out of 10 rats died before catheter evaluation, exhibiting a lower survival rate compared with MMC rats receiving a single ASC injection (MMC-single group) (MMC-single vs. MMC-double, *P* = 0.06; Fig. 5B). In contrast, no deaths were observed during the experimental period in the saline rats, regardless of whether they received one (saline-single group) or two ASC injections (saline-double group). Among the five surviving rats in the MMC-double group, food intake was significantly lower compared with the MMC-single group; however, no significant differences were observed in body weight, RVSP, dp/dt max, or Fulton index (Fig. 5C–E). Furthermore, all parameters in the saline-double group, including body weight, food intake, RVSP, dp/dt max, and Fulton index, were comparable to those in the saline-single group (Fig. 5C–E). To elucidate the cause of the reduced survival rate subsequent to the second ASC injection, the pulmonary condition in MMC rats underwent histological evaluation one day after the second injection. Histological analysis revealed emboli composed of aggregates of nuclear-stained cells within the pulmonary microvasculature (Fig. 5F).

## Discussion

Our study yielded the following three major findings in a PVOD rat model: (1) ASC transplantation inhibited pulmonary microvascular muscularization; (2) ASC transplantation ameliorated right ventricular hypertrophy; and (3) two injections of ASCs increased the mortality rate of the rat model, indicating that the prognosis may have worsened depending on the administration method.

In this study, allogeneic ASC transplantation substantially suppressed vascular smooth muscle cell proliferation in the pulmonary microvessels of the PVOD rat model. In an affected pulmonary arterial smooth muscle cell in familial pulmonary arterial hypertension (PAH), the genetic mutation of BMPR2 disrupts BMP signaling, which results in an imbalance between apoptosis and cell proliferation, thereby causing vascular remodeling [17]. Recently, it has been reported that elevated TSP1–CD47 signaling contributes to the hypertrophy of pulmonary arterial smooth muscle cells in pulmonary hypertension [18]. While the mechanism of PVOD remains unclear, vascular smooth muscle cells have also been found to be involved [15]. In our study, the PVOD rat model increased vascular smooth muscle cell area; thus, inhibition of vascular smooth muscle cell proliferation was found to be conducive to the treatment mechanism of ASC transplantation. We had previously demonstrated that MSC sheets transplanted onto the arterial adventitia induced vascular re-endothelialization and inhibited vascular smooth muscle cell proliferation in a rat model of arterial injury [14]. Factors such as VEGF, angiopoietin, and HGF from MSCs promote vascular re-endothelialization, which may exert an indirect effect on the aforementioned process [19]. As can be seen from Fig. 2B, intravenously injected ASCs were trapped within the lung. The paracrine factor of MSCs released within the pulmonary microvasculature would directly act on its luminal side microvasculature, facilitating vascular re-endothelialization and inhibiting vascular smooth muscle cell proliferation. Recently, mesenchymal stem cell extracellular vesicles (EVs) were reported to have numerous direct therapeutic targets in vascular smooth muscle cells, which also resulted in the suppression of intimal hyperplasia and inflammation [20–22]. For example, Wang et al. reported that abundant miR-125b in mesenchymal stem cell-derived exosomes inhibited neointimal hyperplasia in rat carotid arteries by suppressing myosin 1E expression as well as vascular smooth muscle cell proliferation and migration [23]. Future research should also explore the inhibitory effect of MSC-derived EVs on vascular smooth muscle cell proliferation.

The results of this study indicated that despite the reduction in pulmonary microvascular wall thickness, the overall state of pulmonary hypertension did not improve. Luo et al. reported that ASC transplantation alone substantially promoted a decrease in pulmonary artery wall thickness in monocrotaline-induced rats with pulmonary hypertension compared with the untreated group; however, the mean pulmonary artery pressure did not significantly decrease [24], which is consistent with our results. They also demonstrated that combination therapy with ASCs and adiponectin alleviated pulmonary hypertension [24]. These results and those of our study suggest that therapy with ASCs alone has limited effectiveness. Another possible reason is that the PVOD model had extensive lesions involving the arteries, veins, and capillaries, which may have caused embolization of ASCs and consequently offset their effects.

We also found that ASC transplantation reduced the right ventricular weight ratio in the PVOD model despite the lack of such a reduction in the RVSP. This improvement could be explained by the reduced right ventricular cardiomyocyte hypertrophy, as right ventricular tissue fibrosis remained unchanged. Generally, cardiac hypertrophy develops as a result of wall stress adaptation caused by hemodynamic load, and progression to pathological hypertrophy leads to heart failure [25]. Our results indicated that MMC-exposed rats had a more severe form of cardiac hypertrophy with higher RVSP, whereas this correlation between RVSP and right ventricular hypertrophy was not evident in ASC-transplanted rats. This suggests that there were rats that did not develop right ventricular hypertrophy despite their high RVSP, implying that ASC transplantation enabled the right ventricle to tolerate pressure-overload conditions without hypertrophy.

Previous studies have reported the beneficial effects of MSCs on myocardial hypertrophy. For instance, Gopinath et al. reported that intravenously administered human umbilical cord blood-derived stem cells reversed doxorubicin-induced pathological left ventricular hypertrophy [26]. In addition, Liu et al. reported that intramyocardial injection of human umbilical cord-derived mesenchymal stem cells promoted local CD4 + T-cell migration, angiogenesis, and suppressed hypertrophy following myocardial infarction [27]. Although pulmonary hypertension and myocardial infarction have different mechanisms of development, right ventricular ischemia had been implicated in right ventricular failure associated with pulmonary hypertension [28, 29]; a similar mechanism may also be involved in right ventricular hypertrophy. Our results showed a trend toward lower levels of NT-proBNP. This suggests that ASC transplantation is a cardioprotective measure in the PVOD rat model [16, 30]. Further studies are warranted to elucidate the molecular mechanisms by which ASCs hinder right ventricular hypertrophy and provide myocardial protection without mediating pressure unloading in pulmonary hypertension. Moreover, right ventricular failure, an important prognostic factor, does not appear to be caused by pressure overload alone [28, 31, 32], and the direct effect of ASCs on the right ventricle may provide insights into new therapeutic strategies aimed at right ventricular protection, different from the currently available pulmonary vasodilators utilized pulmonary hypertension.

Existing knowledge on the optimal dose and timing of MSC administration remains controversial. Contrary to expectations, the double-injection treatment using ASCs in the PVOD rat model resulted in a lower survival rate compared with single-injection treatment. Most previous studies on the intravenous administration of cell suspensions for pulmonary hypertension models have utilized single-injection protocols [24, 33–36]. In contrast, this study demonstrated that double-injection treatment in the PVOD rat model led to significant adverse effects, which were not observed in double-injection treatment applied to healthy rats (Fig. 5B). Moreover, the double-injection treatment for the PVOD model did not yield additional therapeutic benefits compared with single-injection treatment in the surviving rats. Tatsumi et al. reported that the tissue factor expressed on the cell membrane of MSCs induced the development of intravascular thrombosis, and dose increase was positively correlated with the survival rate [37]. As regards healthy rats, we found that a single intravenous injection of ASCs led to an even distribution in the lungs, but body weight and food intake did not change during the course of the study. Although dp/dt max, a measure of right ventricular contractility, decreased, RVSP did not increase at week 4 (Fig. 3B and E). Therefore, considering that the administration of ASCs to healthy rats does not result in adverse effects, it is unlikely that the procoagulant activity of ASCs alone is responsible for the severe adverse effects observed in the present study.

Another possible explanation is that the endothelial damage caused by MMC, which has been clinically reported to induce thrombotic microangiopathy [38, 39]. As demonstrated in Fig. 5F, in addition to the increased total number of transplanted cells due to the double-injection protocol, the vascular injury inflicted by MMC likely made the vasculature more susceptible to pulmonary and systemic embolisms, ultimately leading to decreased survival rates. In PVOD, where pulmonary microvascular injury is the main component of the pathogenesis, repeated intravenous administration or administration of more than a certain number of MSCs may lead to severe adverse events due to pulmonary embolism. In a clinical setting, the symptoms of pulmonary hypertension are similar across classifications, and the diagnosis of this rare type of PVOD ultimately depends on its histopathology [40, 41]. PVOD accounts for 5–10% of the cases of initially diagnosed idiopathic PAH [3]. This suggests that cell-based therapy for patients with pulmonary hypertension should be administered with caution.

One limitation of this study is that the molecular pathways by which ASCs confer therapeutic benefits on the lung and heart remain unclear. Although CD47—previously reported to be up-regulated in rodent models of pulmonary hypertension [18]—tended to increase in a subset of animals in our PVOD model (Fig. S5), the change did not reach statistical significance. Consequently, we could not confirm whether ASCs act through the TSP1–CD47 axis. Future investigations should therefore examine TSP1–CD47 signaling in greater detail and explore additional molecular mechanisms, as elucidating these pathways will be essential for developing effective therapies for the PVOD.

## Conclusion

This is the first study to investigate the effect of ASC transplantation on an animal model of PVOD. The results indicated that allogeneic ASC transplantation significantly attenuated pulmonary microvascular muscularization in the PVOD model. Despite the absence of alleviation in pulmonary hypertension, right ventricular hypertrophy became markedly less pronounced. Conversely, the double-injection treatment was not more effective than the single-injection treatment and increased the mortality rate of the rat models. Although allogeneic ASC transplantation has shown some efficacy in treating PVOD and consequent right ventricular failure, it should be carefully considered owing to the risk of side effects depending on the administration method.

## Electronic supplementary material

Below is the link to the electronic supplementary material.

Supplementary Material 1

Supplementary Material 2

Supplementary Material 3

Supplementary Material 4

Supplementary Material 5

Supplementary Material 6

Supplementary Material 7

## Data Availability

All data supporting the findings of this study are included within the article (and its supplementary files). Additional data are available from the corresponding author on reasonable request. Sequencing data was not used.

## Abbreviations

ASCs: Adipose-derived mesenchymal stem cells
αSMA: α-smooth muscle actin
EVG: Elastica-van Gieson
EVs: Extracellular vesicles
FBS: Fetal bovine serum
HE: Hematoxylin-eosin
MMC: Mitomycin C
MSC: Mesenchymal stem cells
NT-proBNP: N-terminal pro-brain natriuretic peptide
PBS: Phosphate-buffered saline
PVOD: Pulmonary veno-occlusive disease
RVSP: Right ventricular systolic pressure
