# Supplementary figures and images for: Intravenous mesenchymal stem cell transplantation mitigates pulmonary vascular remodeling but poses dose related risks in a pulmonary veno-occlusive disease model
Source: Stem Cell Res Ther. 2025 May 28;16:258. doi: 10.1186/s13287-025-04400-8 (PMC12121274; doi:10.1186/s13287-025-04400-8)

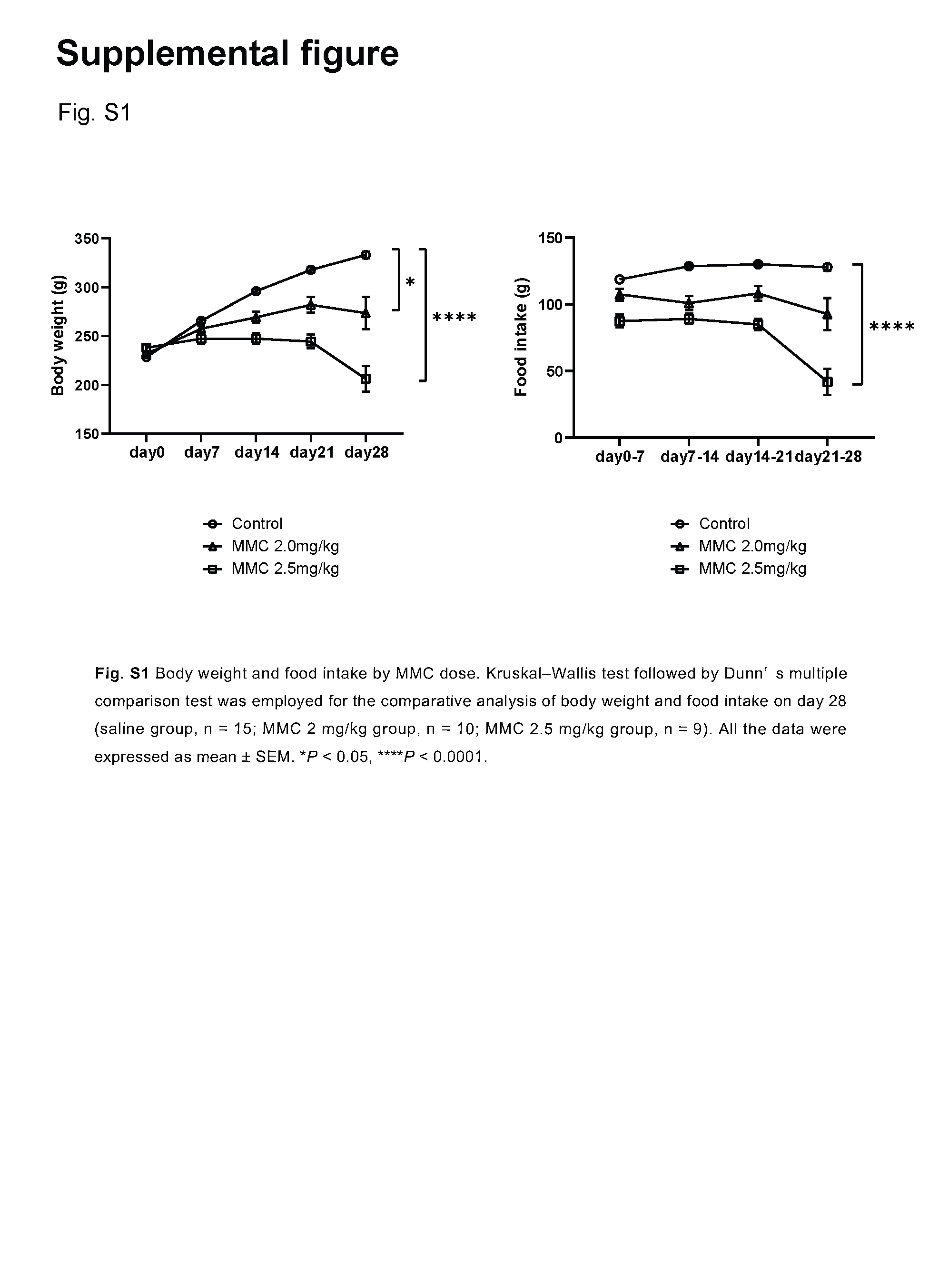

Supplement: Supplementary file 1 — Supplementary Material 1 [file 13287_2025_4400_MOESM1_ESM.tif]

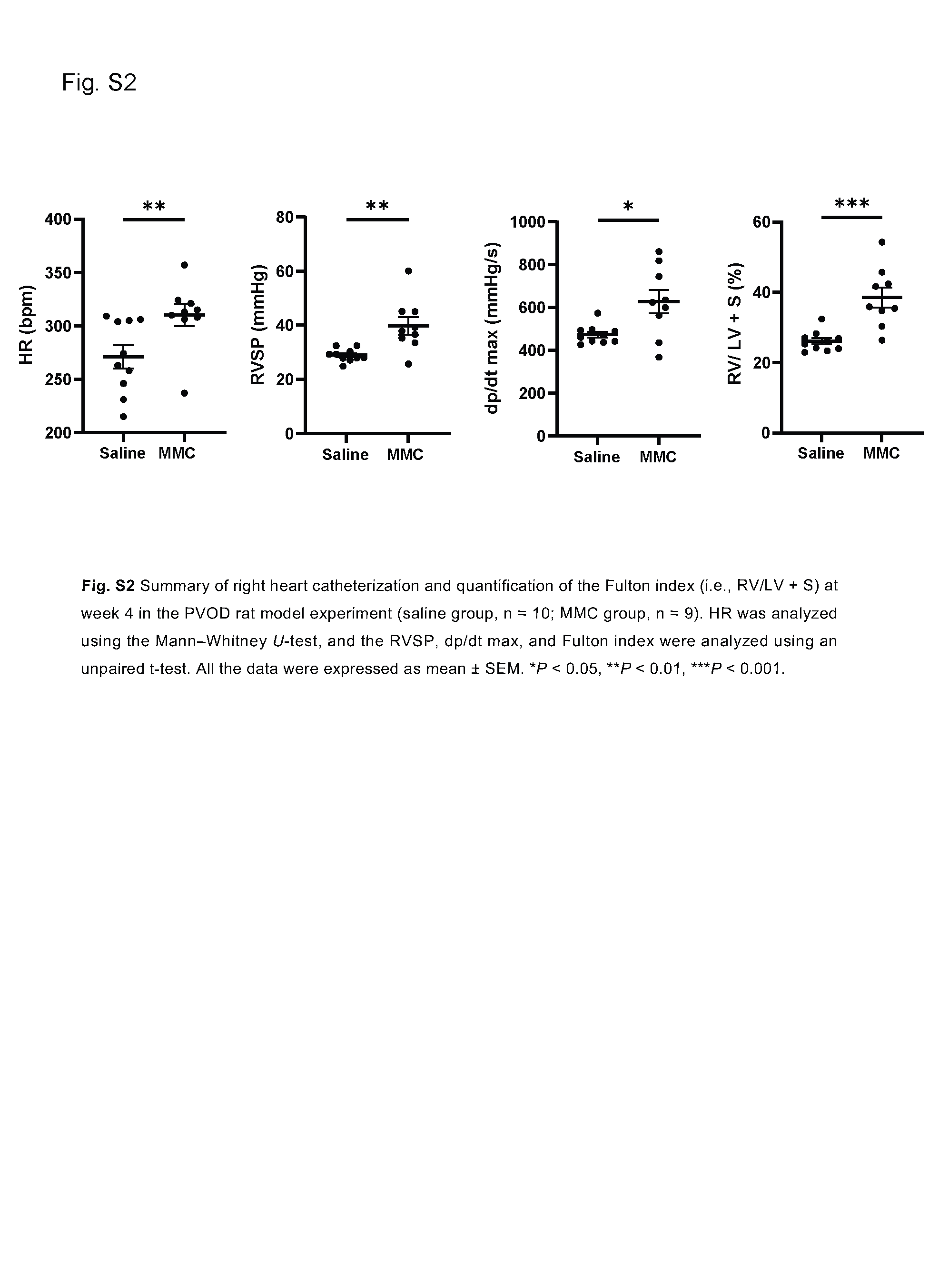

Supplement: Supplementary file 2 — Supplementary Material 2 [file 13287_2025_4400_MOESM2_ESM.tif]

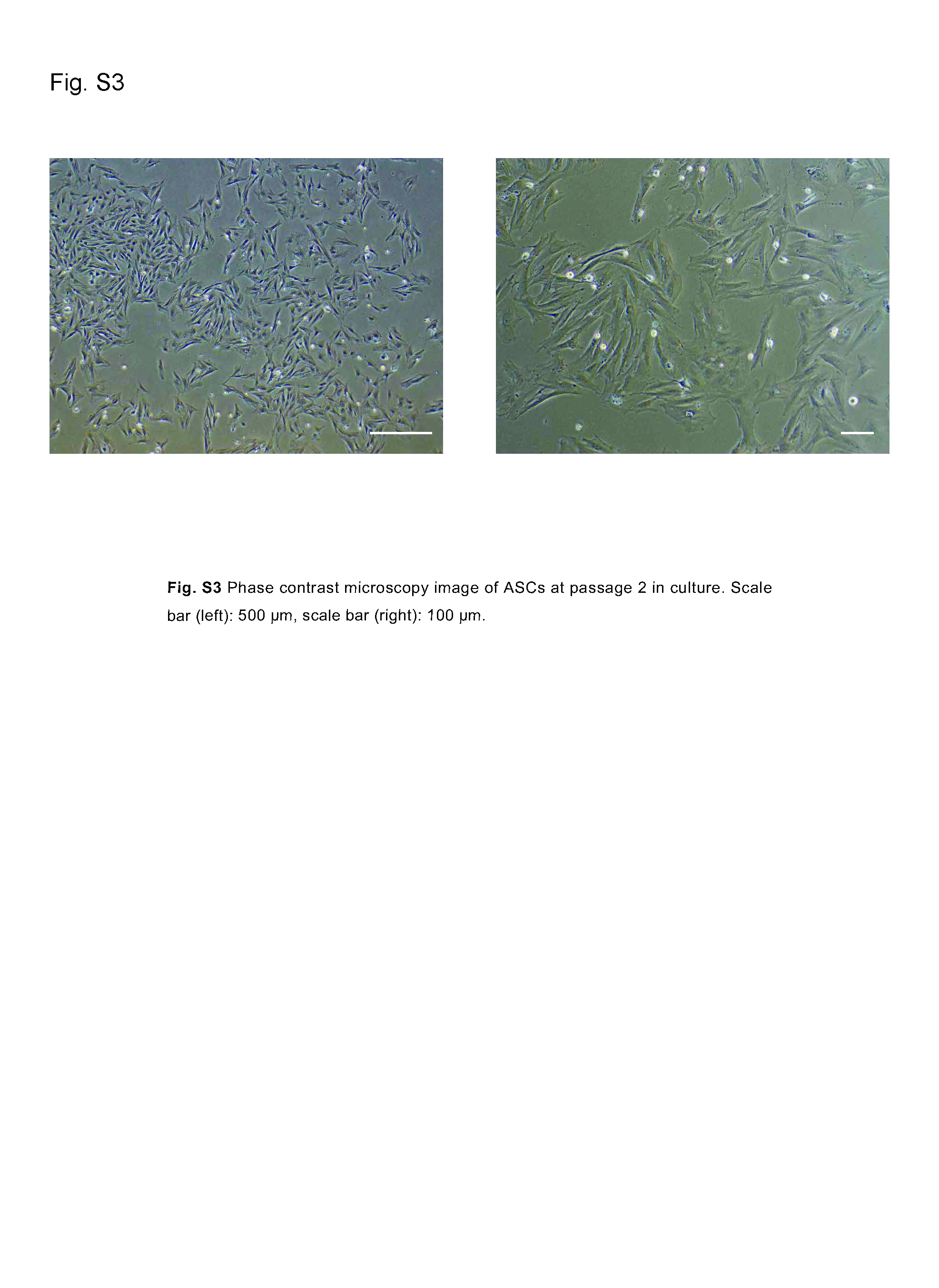

Supplement: Supplementary file 3 — Supplementary Material 3 [file 13287_2025_4400_MOESM3_ESM.tif]

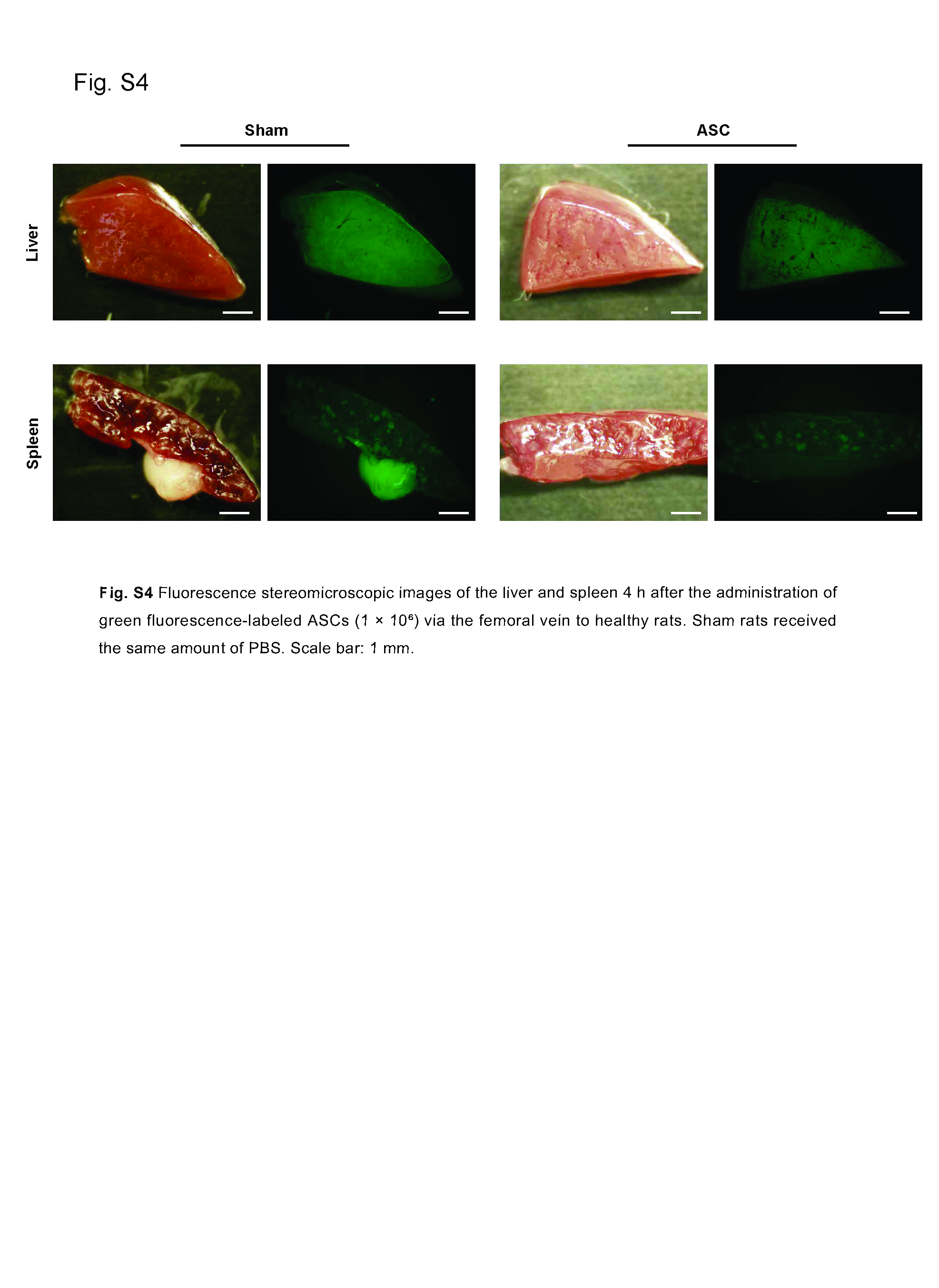

Supplement: Supplementary file 4 — Supplementary Material 4 [file 13287_2025_4400_MOESM4_ESM.tif]

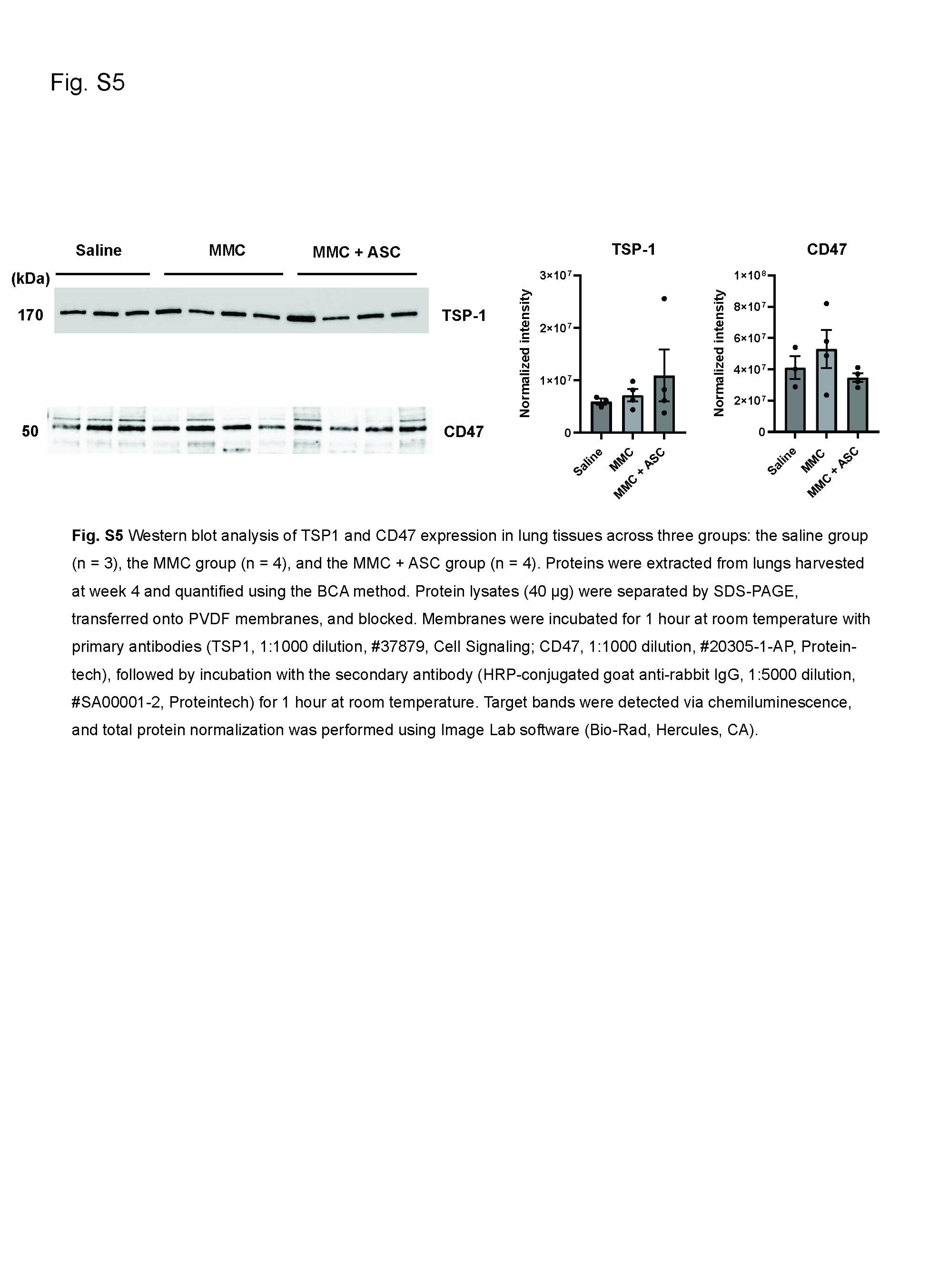

Supplement: Supplementary file 5 — Supplementary Material 5 [file 13287_2025_4400_MOESM5_ESM.tif]

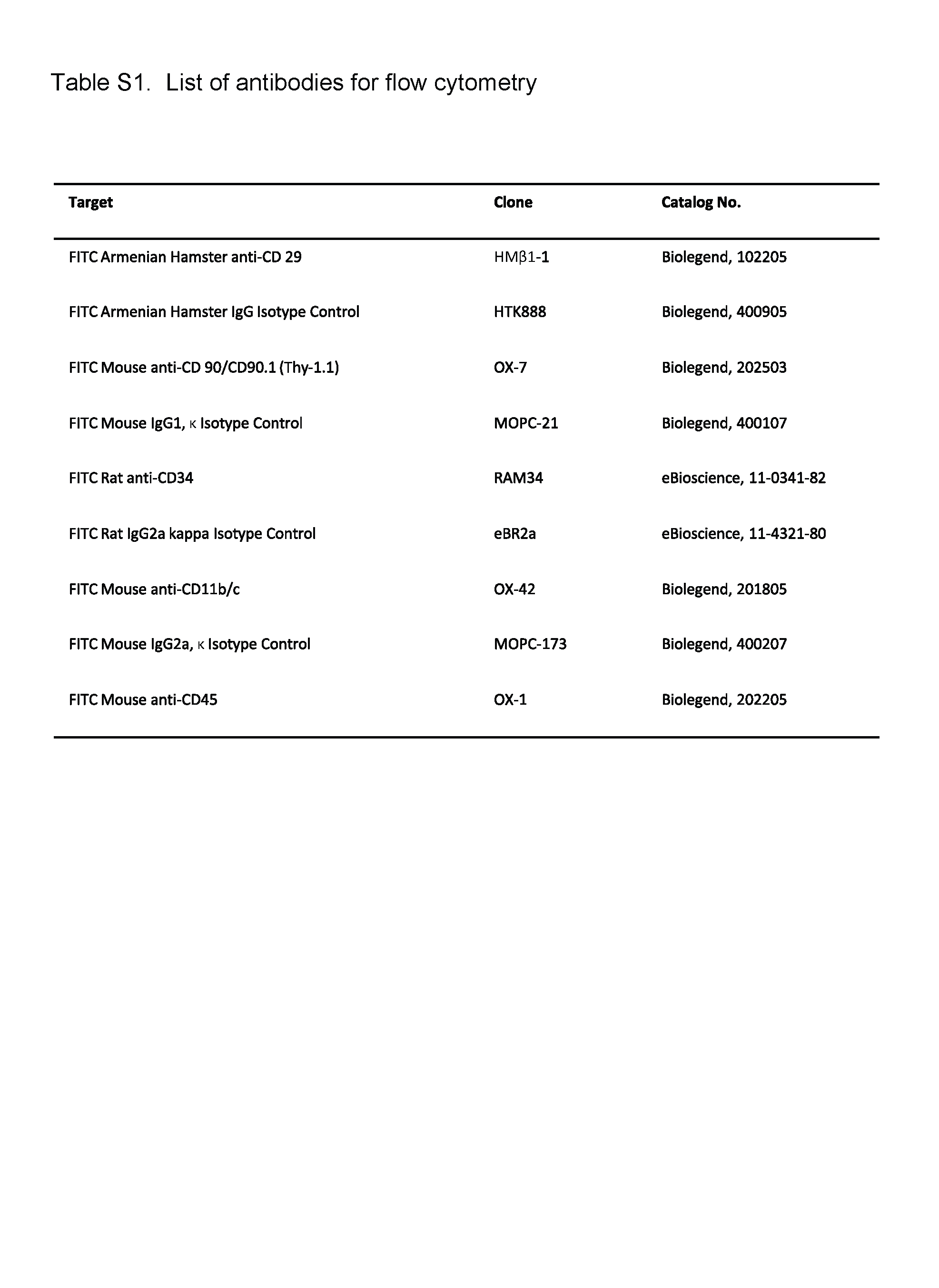

Supplement: Supplementary file 6 — Supplementary Material 6 [file 13287_2025_4400_MOESM6_ESM.tif]
